# Coping strategies employed by older Nepalese migrant women to manage their mental distress in the UK: A qualitative research

**DOI:** 10.1371/journal.pone.0310832

**Published:** 2024-12-12

**Authors:** Lalita Kumari Sah, Rajeeb Kumar Sah, Devendra Raj Singh, Rochelle A. Burgess

**Affiliations:** 1 Faculty of Health Studies, University of Bradford, Bradford, United Kingdom; 2 School of Human and Health Sciences, University of Huddersfield, Huddersfield, United Kingdom; 3 Institute for Global Health, University College London, London, United Kingdom; Patan Academy of Health Sciences, NEPAL

## Abstract

**Introduction:**

Mental health and wellbeing is a global public health concern. However, there is limited evidence on managing the mental health needs of the Nepalese migrant population in the UK. This paper is focused on exploring coping strategies employed by older Nepalese migrant women in managing their mental distress.

**Methods:**

A qualitative study informed by a narrative approach was conducted among twenty Nepalese older women living in London. Interviews were analysed using thematic analysis.

**Results:**

Findings identified three major coping strategies used by Nepalese older women: i) Engaging others to access human, social and economic resources in problem focussed strategies; ii) Using emotion-focussed strategies through drawing on human and social resources; and iii) Employing emotion-focussed strategies through prayer and acceptance. Each strategy reflected the strategic use of existing resources, highlighting a strong sense of ownership over their mental wellbeing.

**Conclusion:**

Nepalese women used both problem-focused and emotion-focused coping strategies to manage their mental distress while living in the UK. However, they had poor awareness of the availability or potential benefits of mental health services in managing their distress and were not able to identify their everyday survival as strengths. We assert social interventions that build on women’s abilities are essential to promote mental health and wellbeing.

## Introduction

According to the World Health Organisation (WHO), mental health includes an individual’s ability to cope with stresses in life [1]. Coping strategies often vary depending on gender, age, sociocultural and economic positions. From a gender perspective, evidence from high-income countries such as the United Kingdom suggests that women are more likely to use ruminative emotional coping styles, which focus on the acceptance of circumstances linked to the causes of their mental health problems [2]. Drawing on religious and cultural practices, for example, prayer is the most common coping strategy when faced with distressful situations among South Asian women, which differentiates them from the White British population in the UK, who are likely to seek professional help [2–4]. Given the socioeconomic challenges that shape the lives of older Nepalese women, including migration status, widowhood, and poverty, such studies highlight that coping strategies must be understood within the realities of everyday lives and experiences of the communities. Given findings across various settings that highlight how mental well-being is linked to these wider structural realities, and there is also a need to reflect on coping with these environments that contribute to overall efforts to promote mental well-being and manage mental distress [5]. The term ‘‘mental distress” is used in this paper as a term that acknowledges the troubling or adverse experiences as used in line with the previous literature [5, 6].

Previous studies indicate that older Nepalese women’s mental wellbeing is also heavily influenced by the intersections of a range of social challenges, such as economic hardship, family/relationship problems, and adjustment to the physical environment (i.e. weather patterns) in the UK [7, 8]. While many studies acknowledge that South Asian communities, including Nepalese communities, have low levels of awareness and uptake of mental health services in the UK [4, 8, 9], very few studies have explored indigenous coping strategies used by these migrant communities to tackle their mental health needs [2, 10]. However, none of these studies have directly explored the coping strategies employed by older Nepalese migrant women in the UK. According to the recent census in 2021, the current Nepalese ethnic population in the UK is over a hundred thousand, and the number of women 60 years and over is 5849 [11]. Hence, it is important to explore the issues experienced by this population group, given the increasing number of older women in this population group, so that appropriate support can be considered in the policy and practices to address their needs. In this study, we aim to explore coping strategies employed by older Nepalese migrant women in managing their mental distress. Under mainstream mental health paradigms, coping is often linked to strategies attempting to an effort to manage the demands created by stressful events [12]. Seminal work by Folkman and Lazarus categorised coping strategies as either problem-focused or emotion-focused [13]. Problem-focused strategies aim to solve the problem or reduce mental stress by taking direct action and seeking assistance. In contrast, emotion-focused strategies aim to manage distress associated with the situation without changing the source of the stress. Since then, these coping approaches have been applied by several studies, such as the spouse of the military in the US [14] and gender differences in the outcomes of coping in education [14, 15]. However, in our understanding, none of the studies have applied the concept to older Nepalese migrant women; this is the first research of its kind.

## Methods

### Study design

Our research focused on the coping strategies employed by older Nepalese migrant women in managing their mental distress and the concept of intersectionality given the intersecting risks they face linked to gender inequality, age and ethnic minority status in the UK [16]. A qualitative approach enabled us to generate an understanding of the local meaning of mental health issues and processes of coping strategies employed by older Nepalese women from their perspectives [17]. This aligns with the philosophical understanding of constructivism [18]. The face-to-face interviews in this research were informed by a narrative approach. Given that mental health is a sensitive and complicated topic to explore, particularly among groups with limited biomedical understandings and high stigma in relation to medicalised framings of mental distress, a narrative approach facilitated participant ownership of the data collection process through establishing narrative and organising major life events in the form of stories, which has been argued as a universal and accessible way to make meaning of those events to reveal the information [19].

### Setting and data collection

Purposive sampling [20] was applied to select research participants because of study’s intention to explore a specific intersection of age, gender, and ethnicity within the Nepalese population. The study recruited 20 female participants of Nepalese ethnic origin who were 60 years old and older. Two pilot studies were conducted to get a deeper understanding and finalise the interview schedule, as well as to gain confidence in using prompts during the interviews. It was a coincidence that all the participants selected for this study were the wives (eleven married women) or widows (nine widows) of ex-Gurkhas. Interviews were conducted by the woman researcher, the first author (LKS), in Nepali, who shares Nepalese ethnicity, spoken language, cultural values, and religious beliefs with the research participants. The study was conducted in the London Borough of Greenwich, where 2.6% of the total population of the Borough are Nepali (including Gurkha) ethnic group, which counts 7654, the highest Nepalese ethnic group in London compared to other Boroughs [11, 21]. The inclusion criteria were being older women aged 60 years and over, belonging to the Nepalese ethnic origin and living in the borough, having the ability to provide consent to participate in this research, and having no diagnosable mental illness at the time of data collection. The interviews were conducted in a local park on mutual agreement of the researcher and research participants. Quiet corners of the park were chosen to balance the protection of confidentiality and ensure the comfort of the research participants. This also aligned with women’s daily routines as they often used the park as a safe space for exercise and engaging with other women from the same community. Interviews lasted for an average of fifty minutes. Data for this study were collected between July and August 2015.

### Data analysis

Twenty semi-structured in-depth interviews were conducted and recorded using a digital audio recorder. The data collection process continued until the data saturation of the emergent themes was achieved [22]. The first author (LKS) anonymised the data with pseudonyms and completed the transcription and translation of the interviews to English. No real names of the participants are used in this research, and all the names are pseudonyms. Authors (RKS and DRS), who are also of Nepalese ethnic origin and speak the Nepalese language, reviewed a sample of the transcription and translation together with the first author for validation. The first author (LKS) and senior author (RAB) identified and reviewed the emerging themes from the first three interviews to avoid lone researcher influence in qualitative research [23]. All interview transcripts were analysed using thematic network analysis [24] and NVivo computer software. This approach organises themes systematically and demonstrates a relationship between basic themes, organising themes, and global themes (S1 File). The main categories of code are presented as organising themes in the thematic network tool, which also reflects the concept of seminal work by Folkman and Lazarus in coping strategies [25].

### Ethical aspects

Research information sheets were explained, queries related to the research were answered, and oral consent was obtained from each participant before the start of the interviews. Where lack of education among the participants led to difficulties signing their names on the paper, verbal and written consent was recorded. Ethical approval for the study was obtained from the University Research Ethics Board, London Metropolitan University (ID number: 11.06.2015.02).

In terms of rigour or trustworthiness, Guba and Lincoln assert all research should consider rigour or trustworthiness [26]. We planned and conducted this study considering the four-dimension criteria (credibility, dependability, confirmability and transferability) suggested by Guba and Lincoln to ensure the robustness and trustworthiness of the study. A couple of transcripts and the major findings from the interviews were discussed with two research participants to ensure rigour and trustworthiness, and their comments/suggestions were taken to improve on the other interviews. The supervisor of this study, colleagues at the university and the research committee at the University reviewed the research questions of this study and feedback from them was taken to improve and reword the research questions.

## Results

**Fig 1:**
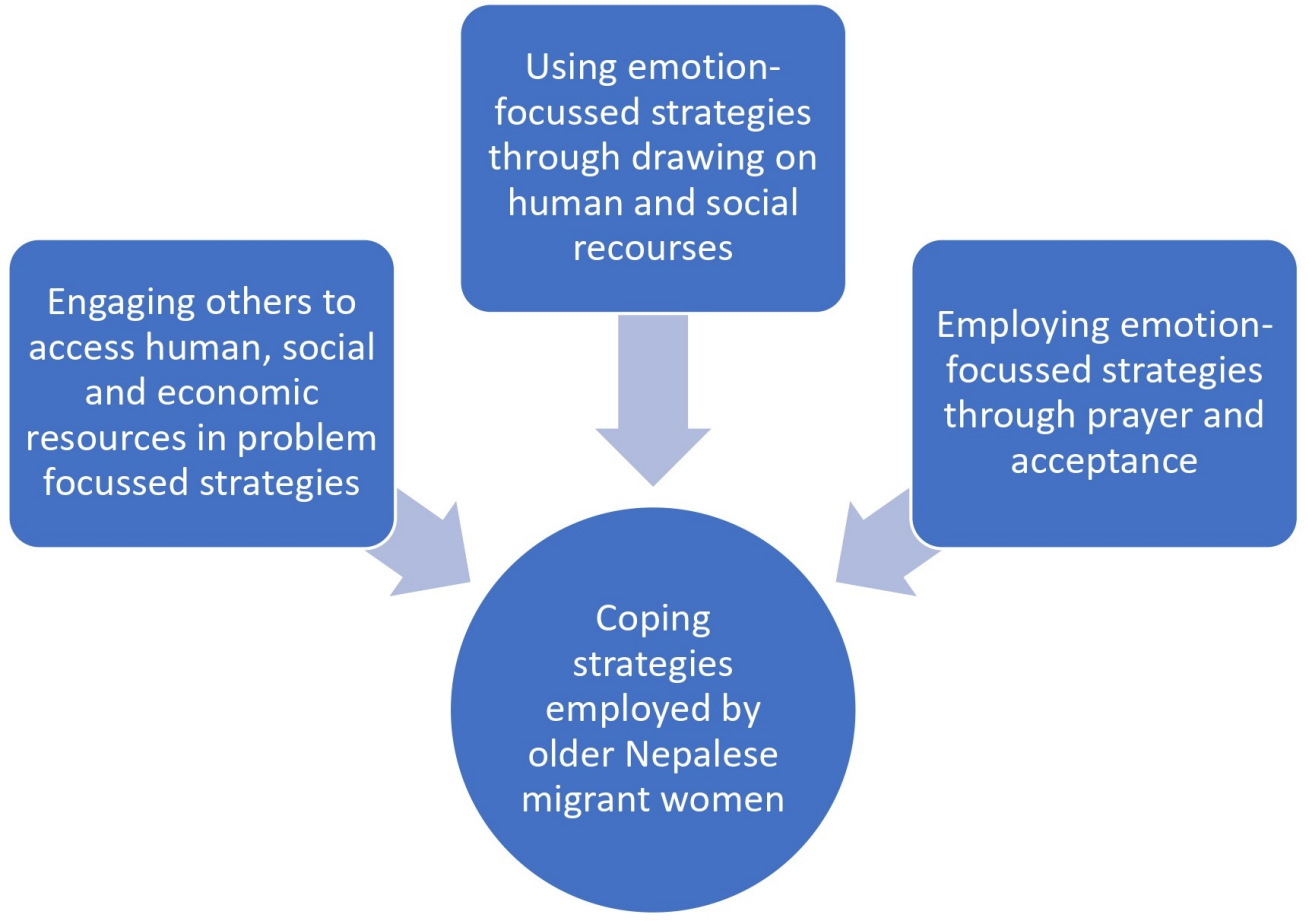
Coping strategies employed by older Nepalese migrant women.

Our analysis identified one global theme and three organising themes describing the coping strategies used by older Nepalese women to address the issues around their mental health and wellbeing: i) Engaging others to access human, social and economic resources in problem focussed strategies; ii) Using emotion-focussed strategies through drawing on human and social recourses and iii) Employing emotion-focussed strategies through prayer and acceptance. We discuss these themes below in separate headings, and the visualisation of the one global theme and three organising themes are presented (Fig 1). All the basic themes are presented as a quote. It is worth noting that when participants were asked about their access to formal mental health services, all women answered that they had never used mental health services in the UK and did not know what they were.

All names of the participants have been changed to protect participant identity. The details of the demographic characteristics of the participants are included in Table 1.

**Table 1:** Participants demographic characteristics.

Interviewee (Participant number and Pseudonym)	Age (Years)	Duration in the UK (Months/Years)	Marital status	Children (total; gender, country of residence)	Living status	Income source
1 (SItta)	68	11 months	Widow	2; residing in Nepal	Living alone	Pension & state benefit
2 (Gita)	80	4months	Married	6; one son in UK, 5 children in Nepal	Living with husband	Pension & state benefit
3 (Santi)	65	4 years	Widow	6; 3 sons and 3 daughters, one daughter in the UK	Living with daughter	Pension & state benefit
4 (Maya)	61	7 years	Married	1; daughter–lives in Nepal	Living with husband	Pension & state benefit
5 (Mayabati)	73	5 years	Married	5; 2 sons in Nepal, One son in Hong Kong, 2 daughters in the UK	Living with husband	Pension & state benefit
6 (Chandra kala)	68	5 years	married	8; 5 sons, 3 daughters; 1 daughter and 3 sons in Hong Kong. 2 daughters and 2 sons are living in the UK.	Living with Husband and youngest daughter	Pension & state benefit
7 (Laxmi)	75	3 years	Widow	4; One son in Hong Kong, one daughter in the UK and 2 children (1 son and 1 daughter) in Nepal	Living alone	Pension & state benefit
8 (Yamuna)	70	10 years	married	5; 3 sons and 2 daughters. One daughter lives in the UK, others are in Nepal	Living with husband and daughter’s family	Pension & state benefit
9 (Radhika)	65	4 months	Widow	5; All are in Nepal	Living alone	Pension & state benefit
10 (Chanta)	61	2 years	Married	4; 3 daughters and one son–all in Nepal	Living with husband	Pension & state benefit
11(Kumari)	72	5 months	widow	2; sons–in Nepal	Living alone	Pension & state benefit
12 (Ganga)	71	3 years	Married	3; 2 sons and 1 daughter—all in Nepal	Living with husband	Pension & state benefit
13(Sangita)	65	6months	Widow	4; 2 sons and 2 daughters–all in Nepal	Living alone	Pension & state benefit
14 (Sobha)	81	3 years	Married	4; 2 daughters and 2 sons, one daughter in America, three in the UK	Living with husband and children.	Pension & state benefit
15 (Nirmala)	68	15 months	Married	7 children—all children are in Nepal	Living with husband	Pension & state benefit
16 (Babita)	70	3 years	Married	One daughter lives in Australia, one son lives in Hong Kong, One daughter lives in the UK	Living with husband and daughter	Pension & state benefit
17 (Ambika)	68	4 years	Married	5; 3 daughters and 2 sons; One daughter lives in the UK, others in Nepal	Lives with husband	Pension & state benefit
18 (Rita)	83	2.5 years	Widow	5; Had 5 sons, 2 died. One lives in Nepal, one lives in Hong Kong, one lives in the UK	Lives alone	Pension & state benefit
19 (Kabita)	70	4 years	Widow	3; 1 son and 2 daughter—all in Nepal	Living alone	Pension & state benefit
20 (Parbati)	73	2.5 Years	Widow	4; 2 sons and 2 daughters–all are in Nepal	Living alone	Pension & state benefit

### i) Engaging others to access human, social and economic resources in problem-focused strategies

Older women in this study who were married and living with their husbands or widows who were living alone relied on other people in their family and the community to solve their problems related to health issues, housing, and finance and seek advice from others to make their daily life easier in the UK. Nepalese older women who lived with their husbands relied on their husbands for assistance in their day-to-day activities because of the language issue in the UK. For example, Maya (61) says:

*Yes, I go to the GP. My husband and family help me. My husband can speak a little bit of English. I am living here with his support*.

*(Maya*, *61)*

This study found that women struggle for financial reasons. The women who had children living in the UK and other countries received financial support from their children while they were living alone in the UK. Babita, who has three children living in other high-income countries such as Australia and Hong Kong, shared her experience:

*The money we get (state benefits and pension) is just enough to eat here and buy clothes. We need to go to Nepal because if something happens in a family, people expect us to be there, such as someone dying or being sick. This money is not even enough to buy good clothes. How is it possible to buy tickets with this money? Sometimes, my son and my daughter buy tickets for me to travel to Nepal*.

*(Babita*, *70)*

Women in this study received help from their neighbours from the same community to solve their health and financial issues. Sita and Radhika were widows living alone in the UK, needed support from their neighbours for their health check-ups because they did not speak the English language and for financial support since they were dependent on state pension and benefits. They shared their experiences as:

*I have high sugar and high blood pressure. I went to the hospital. My neighbour helped me. The hospital gave medicine. Now, I am feeling better*.

*(Sita, 68)*

*I borrowed money from other people in the community to manage daily expenses, and they supported me*.

*(Radhika*, *65)*.

Women who did not have immediate family members in the UK received advice and support from the Nepalese community and local organisations such as Gurkha Welfare. Kumari and Radhika shared their stories:

*Other people from my village in Nepal have been here for the last 2 years. They searched the room for me before I came to the UK. They told me to come here (UK), as it’s easy to live, eat, and survive because of pension and benefits. So, I came here*.

*(Kumari, 72)*

*At first, when I came here, I had some money with me. Then, I got about £1300 from Gurkha Welfare*

*(Radhika*, *60)*

### ii) Using emotion-focussed strategies through drawing on human and social resources

In coping with loneliness, the women seemed active in engaging with people from the same community in the UK and children in the UK and abroad. Women in this research seeked social contact by meeting and spending time together in the park to avoid loneliness. Chandrakala says:

*I come to this park to spend time and enjoy. It gives peace of mind and happiness. We just walk and talk without any reason and sit together in the park, I listen to them, and they listen to me. For happiness, we come here*.

*(Chandrakala*, *68)*

These women were in contact with family through telephone conversations so they did not feel left out. These women regularly spoke to their children over the phone. Chanta says:

*My children call me very often. I also call them. They ask, how are you? Are you healthy or not?*

*(Chanta*, *61)*

The women seemed to have a great desire to travel to Nepal and spend time with their families. In that process, they tried very hard to cut their daily expenses, such as food and other essentials, putting themselves at risk of poor wellbeing and saving some money so that they couldbuy a flight ticket to Nepal. Chandrakala, who had 8 children (1 daughter and 3 sons in Hong Kong, 2 daughters and 2 sons living in the UK), like to meet all her children together as a family in Nepal and shared her experiences as:

*If we want to visit family in Nepal, we need to save money. It is very hard to manage and cut daily expenses here to save money. My children very often go to Nepal from Hong Kong or from the UK. They go for some work reasons or for a holiday. That’s why I am going to Nepal to meet them there*.

*(Chandrakala*, *68)*

### iii) Employing emotion-focussed strategies through prayer and acceptance

Women in this study described how they used prayer in order to cope with their mental distress, such as routine worshipping at home and acceptance of the difficulties they were facing, as an emotional coping mechanism. Their belief that God has created difficulties or stressful situations and that worshipping will provide the strength to overcome this seemed deeply rooted. Women in this study had a hope that worshipping practice will bring positive outcomes and that this difficult situation will change in their favour in the future. Sita shared her rumination practice and beliefs as:

*I believe in God; God has given everything, whether it is happiness or sadness. It is his wish that we will die. Here, we have facilities to eat and live, so there is nothing to worry about. Still, my heart worries, but again, it calms down with the hope that tomorrow, there will be time to live with my family and enjoy time with my children. God has brought us here; I think like that. I always remember God. I believe it is God’s plan to bring us here and make us happy or cry. I am worried, so I worship every morning and evening*.

*(Sita*, *68)*

Women presented a high level of acceptance of their living situation, and they were keen to explore solutions. They preferred to take the blame themselves without complaining about the community support they could have to improve their wellbeing. That may be because they were unaware of the support available. Chandrakala expressed her weakness as:

*I have a problem going around and visiting places on my own as I have language problems. I can’t read or write in the English language. I can be lost anywhere if I go on my own. I am not educated. I can read few words in the Nepali language. That is a sad thing for me. This is my problem, so I can’t say bad thing about this society*.

*(Chandrakala*, *68)*

Women in this study asserted housing as one of the major issues but accepted the housing conditions in which they lived. They seemed ready to adapt to the situation, which could be due to a lack of ability to identify resources and formal support available to them. Maya shared her housing acceptance as:

*When we search for accommodation, we cannot get the house like we want. It is not like what we expect. Not even like we want, but we should live with satisfaction*.

*(Maya*, *61)*

Financial hardship was a major problem women faced in this research. Although they did not receive adequate pension money to live a decent life in the UK, they tried their best to adjust their lives within this financial constraint. Sadly, they accepted that they could not work and earn in their old age and had no alternatives if the money was insufficient. Their acceptance of the situation seemed unusually high, but they did not attempt to explore the other support. Laxmi shared her acceptance of financial constraints as:

*I don’t have other options if the money is not enough, as I can’t work. So, I have to manage and survive with the pension money*.

*(Laxmi*, *75)*

There are several castes and cultures within the Nepalese community, and people differentiate themselves from others in Nepal. But, after coming to the UK, women in this research acknowledged the community as a whole Nepalese population regardless of the caste and culture they present. Kumari and Sangita expressed their opinion as:

*There are many Nepalese people to help us. Moreover, I have relatives as well. So, no problem for me. My caste is THAKURI, there are many THAKURI people in this community*.

*(Sangita, 65)*

*We must behave like every Nepalese as our own people, even though if they are from different places in Nepal. Here, if we see someone Nepalese, we see him or her as our own people. Here, we should not be angry, we should be polite to everyone*.

*(Kumari*, *72)*

It seems the women developed a sense of unity and acceptance that all Nepalese people from diverse backgrounds, languages, and cultures belong to the same community.

## Discussion

Our study highlights that despite living through multiple forms of adversity, Nepalese older women readily deployed individual and collective approaches in accessing human, social and economic resources/support to cope with the social determinants and their experiences of mental distress. To our knowledge, this study is the first of its kind to explore coping strategies employed by older Nepalese migrant women in the UK, viewing psychosocial coping as a process geared at reducing emotional and mental distress during stressful times. We also argue that it is important to consider what resources and strategies women themselves employ in the management of a wide range of challenges linked to their mental wellbeing, particularly in response to structural and emotional determinants of distress. These adaptations highlight the importance of their social networks and cultural practices as pivotal strengths and assets in enhancing their mental health and wellbeing due to their poor understanding and complexities of navigating mental health services.

Our findings confirm the positive benefits of residing in communities with shared ethnic backgrounds and experiences. This is in line with Bhugra and colleague [27] findings that suggest the settling process is eased as the ethnic density increases for migrants from the same community. In that case, evidence suggests that the presence of Nepalese ethnicity is evident in Greenwich [21], and they are more likely to receive from their own community, which was possible when these older women took the opportunity to socialise and contact people from the same ethnicity with whom they share their sociocultural norms and language. Their increasing social contacts, through spending time in the park and talking to each other about their daily lives, counter-acts the distress of isolation for women living alone or away from their children, highlighting that socialising activities positively impact higher life satisfaction among the older Nepalese population [28, 29]. Findings highlighted that the most significant source of social, material, and relational resources to support and improve mental wellbeing in the UK were connected to family, children, and the wider Nepalese community. For older Nepalese women in this study, many benefited from receiving information about the host country and an arrangement of housing before their arrival, contributing to their ability to cope with the situation in the UK. Therefore, we assert that social support network helps older Nepalese women cope with distressing situations and promote their wellbeing, which is in line with the findings from other research, especially studies which report the importance of social support on the quality of life of older people [30, 31]. Some women in our study were fortunate to receive support from family and relatives while they were in the UK. They worked to maintain frequent contact with their family members living elsewhere or in Nepal, citing it as key to maintaining their wellbeing. Such findings are supported by other studies arguing that maintaining a good relationship and contact with people improves wellbeing [29]. Access to financial support from the family positively impacted older women’s mental health and wellbeing. In contrast, separation from family was a leading cause of distress highlighted in previous work [7].

The interactions between poverty, inequality, and emotional wellbeing are well documented [32]. Limited economic resources can be a significant detriment to efforts to cope effectively with stressors in one’s life, particularly among marginalised women [33]. Furthermore, the option to rely on informal support, such as family and friends, in times of stress has been associated with lower socioeconomic status among older people in European countries, including the UK [34]. However, in many instances, these supports are described as limitations rather than positive action in accessing the necessary resources and support. For women in our study, lower socioeconomic status was the most significant challenge, as many women were largely dependent on state benefits and pensions and had limited education. We view women’s abilities to mobilise resources as distinct indicators of their empowerment in coping with and managing distress without accessing structured support. Reliance on wider family members to ensure financial stability can lead to unstable realities in the longer term, which are often shaped by wider geopolitical realities, reducing their access to support. This has been widely reported as a concern in previous studies where remittances highlight relationships between migrant wellbeing and economic realities in home countries [35].

Nepal is known for deeply rooted divisions and differentiations among people based on the caste, culture, religion, and language they speak [36]. We found that Nepalese women in the UK rejected these cultural norms to some extent and accepted people from different castes, cultures, and regions of Nepal under an umbrella as a ’Nepalese community in the UK’. We found that this is unique behaviour in coping with the situation, especially because women have limited alternative options to socialise and build a sense of community due to the constraints of language and cultural barriers in the UK. Our findings also affirm the growing body of evidence citing the value of faith and religion to promote emotional wellbeing. In our study, religious practices and beliefs were one of the main emotions-focused coping strategies the older women use, similar to Indian migrant women in Canada [37]. Engaging in religious activities such as daily prayer and worship brings peace of mind to them and hope for a better situation in the future. This finding affirms previous studies that demonstrate religion is a common coping strategy at the time of psychological distress, which associates positive religious coping with lower depressive symptoms [10].

Our findings support recent calls for expanding definitions of the ’social’ within interventions for the mental health of marginalised communities. There has been growing attention to the value of social interventions in mental health [38], with evidence supporting social prescribing to increase access to relationships that support wellbeing, such as choirs and community gardening [39]. However, these interventions focused primarily on the socio-relational dynamics associated with mental health, ignoring the problematic social-structural dynamics that are driving poor mental health outcomes [40]. Through their abilities, the women in our study established a range of successful coping strategies, leveraging social networks and individual capabilities to promote their wellbeing. This would suggest that for women in this population, gaps in support exist in access to systemic and structural supports around livelihood concerns.

All of our participants had no knowledge or experience of mental health services in the UK. This aligns with earlier research within the Nepalese community, which highlighted a lack of awareness about mental health services and inadequate knowledge about the NHS system to access any health services [8]. The previous literature suggests a low usage of mental health services among migrant communities, despite high levels of mental health issues, whereas the diverse background of migrants creates challenges regarding their utilisation of mental health services in the host country [41, 42] Another reason could be a lack of mental health awareness among these research participants, as they do not necessarily recognise the need for it [9]. The importance of health literacy focusing on physical health is widely accepted, whereas a focus on mental health literacy has been relatively neglected, which could lead to limited access to mental health services among migrant communities in the UK [43]. The language barrier and limited knowledge and understanding of the need and access to mental health and wellbeing services were major reasons for not using mental health services by older Nepalese women in this research. While a study conducted in Nepal showed that Nepalese people were more likely to receive social support for mental health issues from family and friends, our study suggests that for those residing in the UK, this may not always be possible as they live geographically distance from family combined with evidence of high levels of mental health support and low uptake of mental health services [8], suggests more action is needed to promote the mental health of this group. Utilising social networks in a structured form could be a crucial strategy in improving knowledge about mental health services among Nepalese older women in the UK. However, given women’s successful efforts to mobilise social support, bringing services more in line to acknowledge and respond to wider dimensions of social need would help to increase their value to women like those in our study.

## Strengths and limitations

This study presents an understanding of coping strategies employed by older Nepalese women living in the UK, but we did not explore or have any understanding of the presence of mental health disorders among these women. Furthermore, these coping strategies may change according to socioeconomic circumstances, such as family and financial situation. Our findings in this article cannot be generalised to all Nepalese women living in the UK as this study is based on older women (sixty years and over), residing in one London Borough. However, the value and relevance of this research reside in the importance of understanding the coping strategies of the migrant population, which can inform positive building blocks for formalising support to promote good mental health and well-being in the ethnic minority community.

## Conclusion

Our study concludes that older Nepalese migrant women employed both problem-focused and emotion-focused strategies to manage their mental distress while living in the UK. The informal support women were receiving within the family and community was expected to operate in coping with their mental distress instead of formal mental health services. The coping strategies and access to mental health services can significantly promote good mental health and wellbeing. However, the older Nepalese women in this research present a poor understanding of accessing mental health services within the UK’s health system. Therefore, promoting appropriate family and social support combined with adequate access to existing community mental health services would enhance their coping skills and promote mental health and wellbeing. Overall, this paper highlights the need for greater attention and a deep understanding of the sociocultural and economic contexts of older Nepalese women and the ability and resources that underpin their coping strategies for mental health challenges. Further, health and social care service providers should acknowledge these contexts when supporting these women.

## Supporting information

S1 File Basic themes to global themes. (DOCX)

S1 Checklist COREQ (COnsolidated criteria for REporting Qualitative research) checklist. (PDF)
