# Supplementary material for: Coping strategies employed by older Nepalese migrant women to manage their mental distress in the UK: A qualitative research
Source: PLoS One. 2024 Dec 12;19(12):e0310832. doi: 10.1371/journal.pone.0310832 (PMC11637326; doi:10.1371/journal.pone.0310832)
Supplement: S1 File — (DOCX) [file pone.0310832.s001.docx]

S1 File: Basic themes to global themes

| Global Theme | Organising Themes | Basic Themes |
| --- | --- | --- |
| Coping strategies employed by older Nepalese migrant women | Engaging others to access human, social and economic resources in problem-focused strategies | - Husband support for day-to-day tasks - Seeking financial support from the members of the community and Nepalese organisation - Support from the Nepalese community in the UK |
|  | Using emotion-focussed strategies through drawing on human and social resources | - Meeting people from the Nepalese community to pass the time - Sharing problems with friends - Maintaining contact with children back home - Meeting children by travelling to Nepal - Accessing social resources - Saving money for emergencies - Contact with family over phone |
|  | Employing emotion-focussed strategies through prayer and acceptance | - God, faith and practice of praying - Acceptance of problem as own fault and destiny - Sense of community and feeling positive about Nepalese people - Language problem - Accommodation issues - Castes and cultures |
